# Successful retrieval of a lumen-apposing metal stent that had completely migrated into the cavity of a walled-off necrosis

**DOI:** 10.1055/a-2109-1118

**Published:** 2023-07-17

**Authors:** Sho Takahashi, Shigeto Ishii, Akinori Suzuki, Koichi Ito, Ko Tomishima, Toshio Fujisawa, Hiroyuki Isayama

**Affiliations:** Department of Gastroenterology, Graduate School of Medicine, Juntendo University, Tokyo, Japan

Endoscopic ultrasonography (EUS)-guided transgastric drainage of pancreatic fluid collections (PFCs) using a lumen-apposing metal stent (LAMS) is an effective treatment for walled-off necrosis (WON) 1 2 3 . However, misdeployment can occur, which is a severe adverse event 4 5 .

EUS-guided transgastric drainage with a LAMS was performed in a 29-year-old woman, for a WON induced by severe idiopathic acute pancreatitis ( Fig. 1 ). A convex-type echoendoscope (EG-580UT; Fujifilm, Tokyo, Japan) and a 20-mm LAMS (Hot AXIOS; Boston Scientific, Marlborough, Massachusetts, USA) were used. The delivery system of the LAMS was successfully inserted into the cavity of the WON. However, the LAMS migrated into the WON during deployment: after intrachannel deployment, the LAMS was pushed out from the echoendoscope, but because the distance between the echoendoscope and gastric wall was not recognized, the LAMS moved into the WON cavity. EUS revealed that the LAMS was floating in the WON cavity ( Fig. 2 ).

**Fig. 1:**
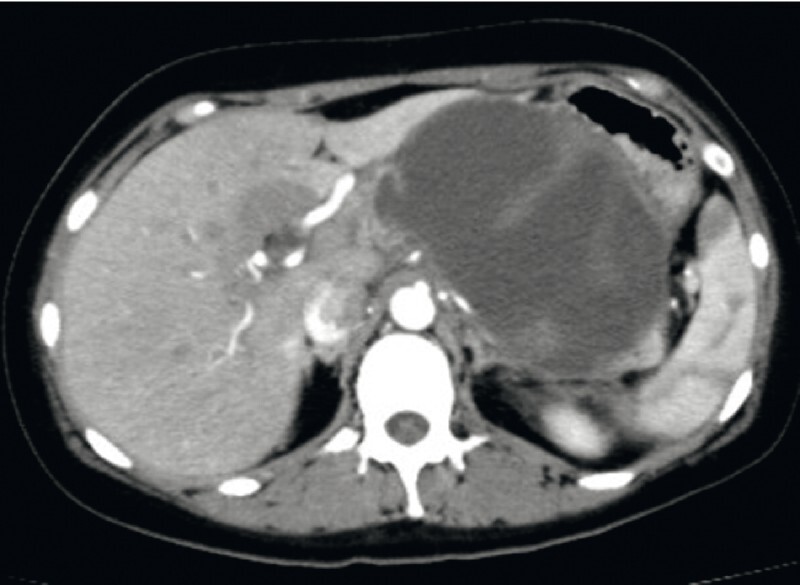
A walled-off necrosis (WON) developed in a 29-year-old woman after severe idiopathic acute pancreatitis.

**Fig. 2:**
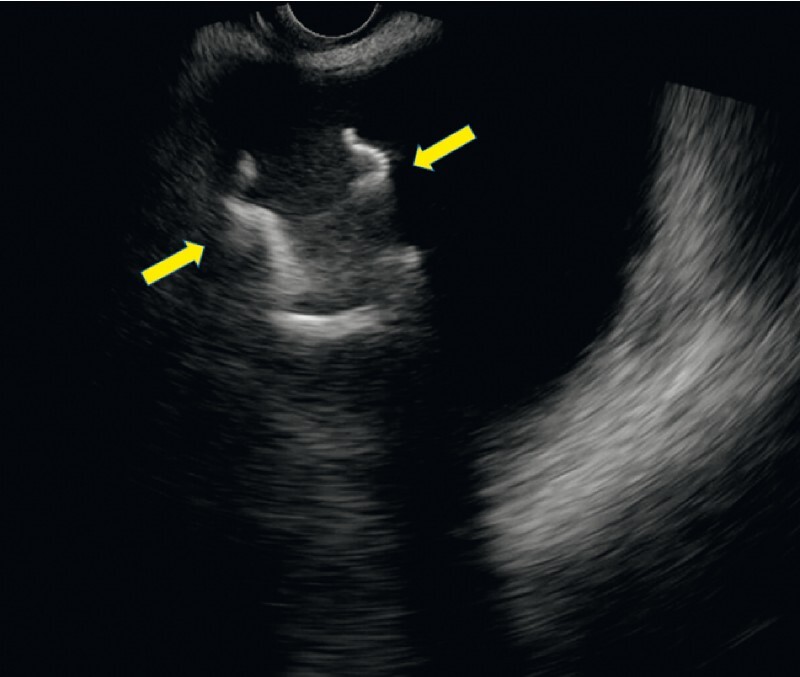
Endoscopic ultrasonography (EUS) shows the migrated lumen-apposing metal stent (LAMS) (yellow arrows) floating in the cavity of the WON.

An additional LAMS was successfully placed in order to remove the migrated LAMS. After balloon dilation (GIGA 14–16-mm; Century Medical Devices, Tokyo, Japan) of the LAMS, a 9.8-mm-diameter forward-viewing endoscope (EG-580RD; Fujifilm) was inserted into the WON cavity through this additional LAMS and the migrated LAMS was observed ( Fig. 3 ). The migrated LAMS was successfully retrieved through the scope by using a snare to grasp the center of the saddle part, without any adverse event ( Video 1 ).

**Fig. 3:**
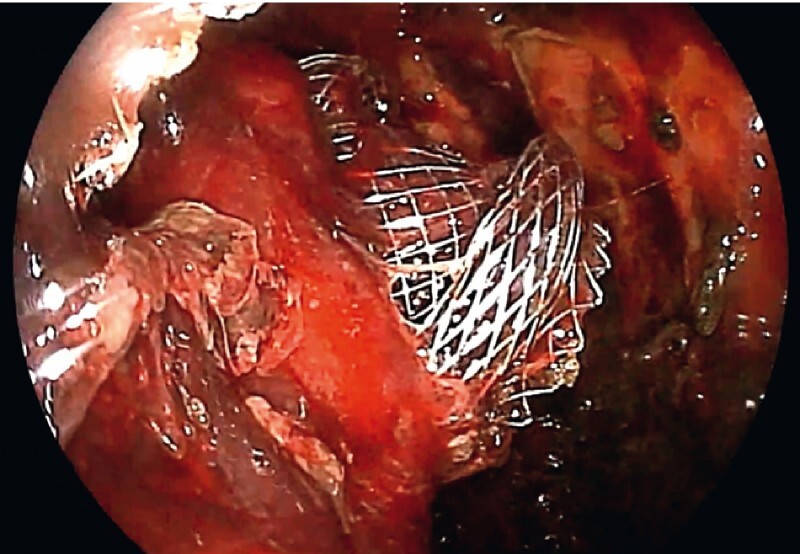
The migrated LAMS was found within the WON.

**Video 1** Endoscopic retrieval of a migrated lumen-apposing metal stent (LAMS) with a forward-viewing endoscope from a walled-off necrosis cavity, following endoscopic ultrasonography (EUS)-guided placement of an additional LAMS.


The use of an additional LAMS was an effective salvage procedure to endoscopically remove a LAMS that had migrated into a WON cavity during an EUS-guided attempt at transgastric drainage of the WON.

Endoscopy_UCTN_Code_CPL_1AL_2AD

## Notes

Endoscopy E-Videos

https://eref.thieme.de/e-videos
*E-Videos* is an open access online section of the journal *Endoscopy* , reporting on interesting cases and new techniques in gastroenterological endoscopy. All papers include a high-quality video and are published with a Creative Commons CC-BY license. Endoscopy E-Videos qualify for HINARI discounts and waivers and eligibility is automatically checked during the submission process. We grant 100% waivers to articles whose corresponding authors are based in Group A countries and 50% waivers to those who are based in Group B countries as classified by Research4Life (see: https://www.research4life.org/access/eligibility/ ). This section has its own submission website at https://mc.manuscriptcentral.com/e-videos
